# Negative Impact of 25-hydroxyvitamin D Deficiency on Breast Cancer Survival

**DOI:** 10.31557/APJCP.2019.20.10.3101

**Published:** 2019

**Authors:** Somchai Thanasitthichai, Aree Prasitthipayong, Krittika Boonmark, Wichai Purisa, Kamolchanok Guayraksa

**Affiliations:** 1 *Breast Division, Department of Surgery,*; 2 *Department of Research, *; 3 *Department of Pathological, National Cancer Institute, Bangkok, Thailand. *

**Keywords:** Vitamin D deficiency, 25(OH)D, breast cancer, survival rate, prognosis

## Abstract

**Background:**

Low 25-hydroxyvitamin D [25(OH)D] levels in serum are associated with breast cancer risk. This study was conducted to determine the impact of 25(OH)D deficiency on survival of breast cancer patients.

**Methods:**

In a retrospective cohort study of 303 patients diagnosed with breast cancer during 2011-2012 at the National Cancer Institute Thailand, all cases were followed up for 7 years. The 25(OH)D was measured by high-performance liquid chromatography (HPLC). Clinical and pathological data were collected. The Chi-square test, Kaplan-Meier and Cox regression model were used to assess the association between 25(OH)D levels and risk of death.

**Results:**

Of the 303 cases aged between 24 and 78 years 51 (16.8%) died during follow-up from any cause. The mean 25(OH)D levels was 25.1±7.54 ng/ml (8.2 – 61.0 ng/ml). Thirty-three patients (10.9%) were stratified as inadequate or deficient group (<16 ng/ml) with mean survival time of 60.65 months compared to 76.24 months in insufficient or sufficient group (≥16 ng/ml). Multivariate analysis adjusted for age, body mass index, stage, lymph node metastases, and immunohistochemical (IHC) findings (*ER*, *PgR*, *HER-2*, *Ki-67* and* P53*) showed that patients with low 25(OH)D levels (<16 ng/ml) at diagnosis had a significantly higher risk of death (hazard ratio = 2.5-2.9) than the group with high 25(OH)D levels (≥16 ng/ml).

**Conclusion:**

A concentration of 25(OH)D below 16 ng/ml was found to be independently associated with poor survival in breast cancer patients, regardless of age, lymph node status, stage or breast cancer subtype. An investigation of potential benefit of 25(OH)D supplements appears warranted.

## Introduction

Breast cancer is the leading female cancer worldwide in both incidence and mortality, with an estimated 1.7 million new cases and over half a million deaths in 2012 (Hu et al., 2018). A great deal of effort has been invested in countering this public health problem with implementation of screening mammography, improvement of access to cancer care and development of anti-cancer protocols. However, problems with poor outcome still remain in many countries.

25(OH)D, a fat-soluble vitamin generated by sunlight exposure or obtained through the diet, plays roles in calcium homeostasis, neuromuscular and immune functions and control of inflammation. Associations of low serum 25(OH)D with cancer incidence and mortality are in line with its anti-cancer properties (Lim et al., 2015), with contributions to regulation of cell growth and apoptosis (Kermani et al., 2011). Epidemiologic studies have generally indicated an inverse association between the 25(OH)D levels and risk of cancers including breast cancers (Goodwin et al., 2009; Gorham et al., 2007; Crew et al., 2009; Bao et al., 2010; Shui and Giovannucci, 2014). Furthermore, breast cancers in patients with low 25(OH)D levels (below 30 or 32 ng/ml) usually have more aggressive clinico-pathological characteristics that result in a poorer prognosis (Palmieri et al., 2006; Goodwin et al., 2009; Thanasitthichai et al., 2015). However, an optimal 25(OH)D cut-off level for prediction of survival outcome under given clinico-pathological conditions has yet to be established. Therefore, we conducted the present retrospective cohort study of 25(OH)D levels in breast cancer patients at the National Cancer Institute, Thailand.

## Materials and Methods

*Study participants*

In this study, 303 patients newly diagnosed with breast cancer between July 2011 and June 2012 were enrolled. All patients were examined for their baseline 25(OH)D levels both before and after treatment. Analyses were carried out with age, BMI, clinical stage, lymph node metastasis, *ER*, *PgR*, *HER-2*, *P53*,* Ki-67* and 25(OH)D levels as variables. The study was approved by the Institutional Review Board and Ethics Committee of NCI, Thailand. Written informed consent was obtained from all patients. 

*Serum collection and 25(OH)D levels assessment*

Approximately 1.5 ml aliquots of serum were collected from 7 ml whole blood samples of each patient and stored at -20ºC until 25(OH)D concentrations were determined by high-performance liquid chromatography (HPLC) with UV detection at the Department of Immunology at NCI, Thailand ((after (Neyestani et al., 2007)). 

*Statistical analysis*

The average of serum 25(OH)D levels was included in analysis, which homogeneity of variance by Cochran and Bartlett’s test were not different. Adopting cut-off points from the literature (Ross et al., 2011) serum 25(OH)D levels were classified as inadequate or deficient when <20 ng/ml (inadequate 12-20 ng/ml, deficient <12 ng/ml), insufficient with 20-30 ng/ml, and sufficient with >30 ng/ml. The impact of 25(OH)D deficiency on breast cancer survival was investigated using the 25(OH)D cut point of 16 ng/ml (calculated from median of IOM definitions for inadequate 25(OH)D, 12-20 ng/ml), above and below which patients were respectively considered to have sufficient and deficient levels. Descriptive statistics were applied with the Chi-square test used to evaluate the significance of differences in parameters between groups. Overall survival was calculated from the date of diagnosis to the date of last follow-up or death from any cause. To compare survival times, the Kaplan-Meier method and log-rank test were employed. Univariate and multivariate statistics were used to estimate hazard ratios (HRs) and 95% confidence intervals (95%CIs) with the Cox’s proportional hazard model. Significance was concluded with p-values < 0.05.

## Results

The mean age for the 303 patients was 50.8±10.5 years (range: 24 – 78 years). Their mean 25(OH)D concentration was 25.1±7.54 ng/ml (8.2-61.0 ng/ml). Thirty-three cases constituted the deficient group (<16 ng/ml) with a mean serum 25(OH)D concentration of 13.3±1.9 ng/ml (8.2-15.6 ng/ml). The remaining 270 (86.0%) patients had a mean value of 26.6±6.7 ng/ml (16.0-61.0 ng/ml). Cases with high 25(OH)D levels (≥16 ng/ml) were mainly early stage or with *HER-2* negative status (p = 0.042 and 0.046, respectively) as detailed in Table 1. 

**Figure 1:**
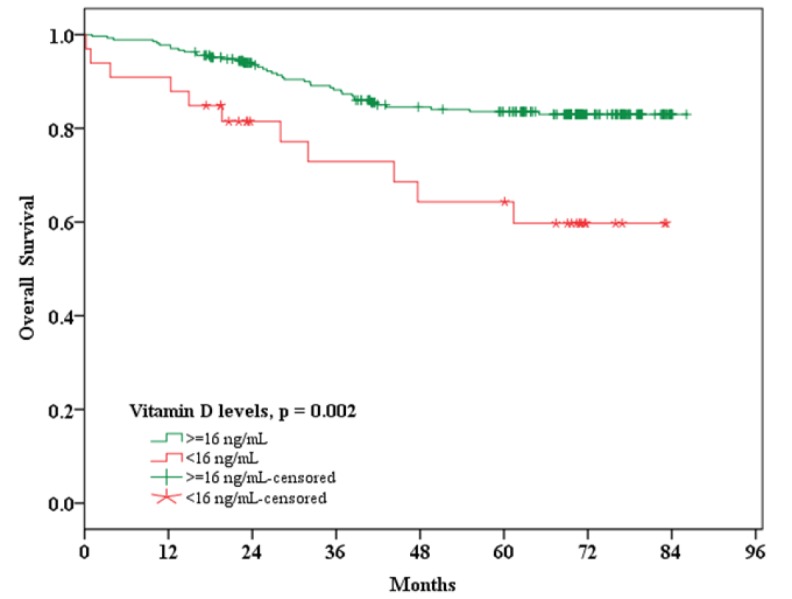
Kaplan-Meier plot for overall survival in breast cancer patients with serum 25(OH)D levels <16 ng/ml versus ≥16 ng/ml

The mean survival time was 74.8 months, with an overall survival (OS) of 81.5% at 60 months. Breast cancer patients in the deficient group (<16 ng/ml) had poorer outcomes for overall survival compared with patients with sufficient 25(OH)D (≥16 ng/ml) (p = 0.002). In the deficient patients with older age, BMI (≥23 kg/m^2^), advance stage, lymph node metastasis, negative for *PgR*, *HER-2*, *P53*, *Ki-67*, and positive for ER showed significantly poorer 5 year-survival rate compared to their sufficient counterparts (p = 0.003, 0.007, 0.024, 0.001, 0.001, 0.001, 0.001, 0.027, and 0.012, respectively) (Table 2). Figure 1 shows Kaplan-Meier plots. Patients with deficient serum 25(OH)D levels had a significantly poorer overall survival than the patients with sufficient serum 25(OH)D levels (p = 0.002).

We conducted univariate and multivariate analysis to determine risk factors affecting breast cancer survival. The multivariate findings after adjustment for age, body mass index, stage, lymph node metastases, IHC findings (*ER*, *PgR*, *HER-2*, *Ki-67* and *P53*) showed that patients with 25(OH)D levels <16 ng/ml at diagnosis had a significantly higher risk of death (hazard ratio = 2.5-2.9) than the group with 25(OH)D levels ≥16 ng/ml (Table 3).

## Discussion

From our analysis, we found that application of a cut-off value of 16 ng/ml for serum 25(OH)D levels is appropriate for division of patients regarding risk of mortality. Our findings are in line with other reports that low serum 25(OH)D has a negative effect on overall and disease-free survival (Ismail et al., 2018; Yao et al., 2017). Moreover, in an additional analysis, by dividing the patients into 3 groups: inadequate (<16 ng/ml), insufficient (16-30 ng/ml), and sufficient (>30 ng/ml) levels, we found that not only the patients with serum 25(OH)D >30 ng/ml had no survival benefit from higher 25(OH)D, but also they had worse survival trend compared to the intermediate level (data not shown). This might imply that the vitamin D supplement might have no additional benefit in patients who are not actually deficient. 

Investigation of prognostic effects of serum 25(OH)D levels in a prospective cohort of 512 women with early breast cancer, revealed those with serum 25(OH)D levels <20 ng/ml had poorer overall survival (HR = 1.73; 95% CI, 1.05-2.86) compared to those with >28.8 ng/ml, on univariate analysis. However, no statistical significance was found after adjusting for age, tumor stage, nodal stage, estrogen receptor status, and histological grading on multivariate analysis (HR = 1.60; 95%CI, 0.96-2.64) (Goodwin et al., 2009). In the study by Lim et al. clinico-pathological data were collected for patients, including serum 25(OH)D levels at diagnosis and at annual follow-up until 2012. Patients with advanced stage disease or older age in the non-deficient group (≥20 ng/ml), showed a significantly better survival compared with the deficient group, suggesting that sustaining optimal serum 25(OH)D levels should be advised for breast cancer patients (Lim et al., 2015). In a Norwegian study, patients who were diagnosed with breast cancer with higher serum 25(OH)D levels (>35 ng/ml) had a significantly decreased risk of breast cancer-specific mortality compared to those with lower serum 25(OH)D levels (<20 ng/ml) (Tretli et al., 2012). In a randomized clinical trial patients with high levels of 25(OH)D prior to chemotherapy had significantly improved overall survival and progression-free survival (Ng et al., 2019).

**Table 1:** Patient Characteristics and 25(OH)D Levels

Characteristics	Total n (%)	Deficient (<16 ng/ml) n (%)	Sufficient (≥16 ng/ml) n (%)	p-value
Age group (years)				0.332
<50	143 (47.4)	13 (39.4)	130 (48.3)	
≥50	159 (52.6)	20 (60.6)	139 (51.7)	
BMI (kg/m^2^)				0.424
<23	128 (43.4)	16 (50.0)	112 (42.6)	
≥23	167 (56.6)	16 (50.0)	151 (57.4)	
Clinical Stage				0.042
Stages I-II	189 (69.5)	16 (53.3)	173 (71.5)	
Stages III-IV	83 (30.5)	14 (46.7)	69 (28.5)	
LN metastasis				0.49
Positive	117 (51.3)	15 (57.7)	102 (50.5)	
Negative	111 (48.7)	11 (42.3)	100 (49.5)	
ER				0.94
Positive	170 (64.9)	19 (65.5)	151 (64.8)	
Negative	92 (35.1)	10 (34.5)	82 (35.2)	
PR				0.776
Positive	129 (49.2)	15 (51.7)	114 (48.9)	
Negative	133 (50.8)	14 (48.3)	119 (51.1)	
HER-2				0.046
Positive	49 (19.2)	6 (22.2)	43 (18.9)	
Equivocal	52 (20.4)	10 (37.0)	42 (18.4)	
Negative	154 (60.4)	11 (40.7)	143 (62.7)	
P53				0.299
Positive	206 (78.0)	24 (85.7)	182 (77.1)	
Negative	58 (22.0)	4 (14.3)	54 (22.9)	
Ki-67				0.225
Positive	238 (93.3)	22 (88.0)	216 (93.9)	
Negative	17 (6.7)	3 (12.0)	14 (6.1)	

BMI, body mass Index; LN, lymph node; ER, estrogen receptor; PR, progesterone receptor; HER-2, human epithelial growth factor receptor-2.

**Table 2:** Five Year Overall Survival Proportions with Patient Characteristics According to the Serum 25(OH)D cut-off

Characteristics	n	5-year survival (%)		p-value
		<16ng/ml	≥16ng/ml	
Vitamin D	303	64.3	83.5	0.002
Age (years)				0.003
<50	143	80.8	89	
≥50	159	54	78.2	
BMI 23 kg/m^2^				0.007
<23	128	81.3	75.1	
≥23	167	51.9	91.9	
Clinical stage				0.024
I-II	189	81.8	92.6	
III-IV	83	42.9	61.3	
LN metastasis				0.001
Positive	117	45	72.2	
Negative	111	87.5	96.5	
ER				0.001
Positive	170	52.1	86.2	
Negative	92	70	77.7	
PgR				0.001
Positive	129	61.9	90.3	
Negative	133	57.1	76.2	
HER-2				0.012
Positive	49	62.5	70.6	
Equivocal	52	75	76	
Negative	154	51.1	88.2	
P53				0.001
Positive	206	65.5	85.7	
Negative	58	50	75.6	
Ki-67				0.027
Positive	238	67.9	82.9	
Negative	17	66.7	85.7	

**Table 3:** Univariate and Multivariate Analyses Evaluating the Baseline Risk Factors that Affect Survival

	Overall survival			
	Unadjusted HRa (95%CI)	p-value	Adjusted HR (95%CI)	p-value
25(OH)D levels stratified by age ^b^				
Insufficient/sufficient (≥16 ng/ml)	Reference		Reference	
Inadequate (<16 ng/ml)	2.626 (1.344-5.130)	0.005	2.474 (1.084-5.644)	0.031
25(OH)D levels stratified by BMI ^c^				
Insufficient/sufficient (≥16 ng/ml)	Reference		Reference	
Inadequate (<16 ng/ml)	2.541 (1.261-5.122)	0.009	2.699 (1.161-6.272)	0.021
25(OH)D levels stratified by Stage^c^				
Insufficient/sufficient (≥16 ng/ml)	Reference		Reference	
Inadequate (<16 ng/ml)	2.138 (1.086-4.207)	0.028	2.429 (1.148-5.139)	0.02
25(OH)D levels stratified by HER-2^c^				
Insufficient/sufficient (≥16 ng/ml)	Reference		Reference	
Inadequate (<16 ng/ml)	2.448 (1.188-5.045)	0.015	2.499 (1.095-5.704)	0.03
25(OH)D levels stratified by LN ^d^				
Insufficient/sufficient (≥16 ng/ml)	Reference		Reference	
Inadequate (<16 ng/ml)	3.190 (1.557-6.534)	0.002	2.493 (1.090-5.704)	0.03
25(OH)D levels stratified by PR ^e^				
Insufficient/sufficient (≥16 ng/ml)	Reference		Reference	
Inadequate (<16 ng/ml)	2.942 (1.494-5.794)	0.002	2.559 (1.113-5.884)	0.027
25(OH)D levels stratified by P53 ^e^				
Insufficient/sufficient (≥16 ng/ml)	Reference		Reference	
Inadequate (<16 ng/ml)	2.972 (1.469-6.014)	0.002	2.518 (1.098-5.773)	0.029
25(OH)D levels stratified by ER ^f^				
Insufficient/sufficient (≥16 ng/ml)	Reference		Reference	
Inadequate (<16 ng/ml)	3.115 (1.581-6.141)	0.001	2.966 (1.399-6.286)	0.005
25(OH)D levels stratified by Ki-67 ^g^				
Insufficient/sufficient (≥16 ng/ml)	Reference		Reference	
Inadequate (<16 ng/ml)	2.325 (1.075-5.031)	0.032	2.462 (1.050-5.773)	0.038

^a^, Hazard ratio; ^b^, Adjusted for LN, ER, and HER-2; ^c^, Adjusted for age, LN, and ER; ^d^, Adjusted for age, ER, and HER-2; ^e^, Adjusted for age, LN, ER, and HER-2; ^f^, Adjusted for age, LN, and HER-2; ^g^, Adjusted for LN and p53”

Mohr et al. also found that high serum 25(OH)D status was associated with lower mortality in patients with breast cancer, recommending that a normal range (30-80 ng/ml) should be maintained with appropriate monitoring (Mohr et al., 2014). In addition, O’Brien et al., (2017) reported high serum 25(OH)D levels and regular 25(OH)D supplement use to be associated with lower rates of breast cancer, especially in postmenopausal women. Similar findings were recently published by Estébanez et al., (2018). From a meta-analysis Kim and Je (2014) concluded that high 25(OH)D status is weakly associated with low breast cancer risk, but strongly associated with better breast cancer survival. Low 25(OH)D levels may be more significantly associated with locally advanced or metastatic breast cancer compared with early stages (Palmieri et al., 2006). Overall, the results support the hypothesis that 25(OH)D supplementation is useful in breast cancer prevention.

However a cohort study of 585 breast cancer survivors higher serum 25(OH)D was associated with improved survival, but the result was not statistically significant and interpretation is difficult (Villaseñor et al., 2013). Furthermore, other results did not support recommendations for vitamin D supplementation to improve breast cancer outcome (Lohmann et al., 2015). In a very recent nationwide, randomized, placebo-controlled trial, with a two-by-two factorial design, 25(OH)D (cholecalciferol) at a dose of 2,000 IU per day did not result in a lower incidence of invasive cancer or cardiovascular event than placebo (Manson et al., 2019).

As a strength of this study, we used a longitudinal cohort. As a possible limitation, to should be mentioned that only a single measurement of serum 25(OH)D levels was made. However, serum 25(OH)D has been reported to remain relatively stable over time, so this may not cause significantly bias (McKibben et al., 2016; Hu et al., 2018). A previous study found some evidence that serum 25(OH)D varies during the calendar year (Fohner et al., 2016).

For explanation of anticancer effects, 25(OH)D can regulate the whole process of tumorigenesis from initiation to metastasis and cell-microenvironment interactions. 1α,25-dihydroxyvitamin D (1α,25(OH)_2_D_3_, calcitriol) roles are mediated by the vitamin D receptor (VDR) (Hu et al., 2018). Regulation of apoptosis, autophagy, inhibits cell proliferation, differentiation, epithelial-mesenchymal transition (EMT), and cell-microenvironment interactions have been proposed (Jeon and Shin, 2018). Metastasis is a complex and multistep process, during which circulating tumor cell (CTC) spread from the primary tumor mass, in the reversible EMT form to the distant organs. Once distant organs are reached, these mesenchymal tumor cells reverse to an epithelial identity via mesenchymal-epithelial (MET) to regain the ability to proliferate (Thanasitthichai et al., 2015).

Furthermore, vitamin D also activates transcription factors of FoxO protein, which control cell proliferation and survival by inducing deacetylation and dephosphorylation, for example, in neuroblastoma cells (Jeon and Shin, 2018). FoxO is a tumor suppressor associated with longevity. There is evidence that 1α,25-dihydroxyvitamin D_3_ (1α,25(OH)_2_D_3_, calcitriol) and FoxO regulate common target genes. VDR is linked with FoxO protein and also regulates the sirtuin 1 (Sirt1) class III histone deacetylase (HDAC) and protein phosphatase 1, providing a molecular basis for cancer chemopreventive actions of 1α,25(OH)_2_D_3_ (An et al., 2010). Apoptosis can be induced by 25(OH)D compound (1α,25(OH)_2_D_3_, EB 1089, and CB 1093) inhibited by Bcl-2 in MCF-7 and T47D human breast cancer cells expressing wild-type and mutant p53, respectively. This finding may indicate potential for treatment of tumors that are resistant to therapeutic agents that are dependent on the activation of p53 and/or caspases (Mathiasen et al., 1999).

In conclusion, our findings suggest that low levels of serum 25(OH)D are significantly associated with poor survival of breast cancer patients. It remains unclear whether adding vitamin D supplement to traditional breast cancer therapy would be a safe way to improve overall survival of breast cancer patients. Further prospective cohort studies and clinical trials are needed for clarification.
